# Supplementary material for: Tailoring chemical composition of solid electrolyte interphase by selective dissolution for long-life micron-sized silicon anode
Source: Nat Commun. 2023 Nov 9;14:7247. doi: 10.1038/s41467-023-43093-6 (PMC10636032; doi:10.1038/s41467-023-43093-6)
Supplement: Supplementary file 1 — Supplementary Information [file 41467_2023_43093_MOESM1_ESM.pdf]

Supplementary Information for

Tailoring chemical composition of solid electrolyte interphase by selective dissolution for long-life micron-sized silicon anode

Yi-Fan Tian[1,2], Shuang-Jie Tan[1], Chunpeng Yang[3], Yu-Ming Zhao[1], Di-Xin Xu[1,2], Zhuo-Ya Lu[1,2], Ge Li[4], Jin-Yi Li[4], Xu-Sheng Zhang[1,2], Chao-Hui Zhang[1,2], Jilin Tang[2,5], Yao Zhao[5], Fuyi Wang[5], Rui Wen[1], Quan Xu[4] and Yu-Guo Guo[1,2]*

1. CAS Key Laboratory of Molecular Nanostructure and Nanotechnology CAS Research/Education Center for Excellence in Molecular Sciences, Institute of Chemistry Chinese Academy of Sciences (CAS), Beijing 100190, P. R. China

2. School of Chemical Sciences, University of Chinese Academy of Sciences, Beijing, P. R. China

3. School of Chemical Engineering and Technology, Tianjin University, Tianjin 300072, P. R. China

4. Beijing IAmetal New Energy Technology Co., Ltd, Beijing, P. R. China

5. CAS Key Laboratory of Analytical Chemistry for Living Biosystems, CAS Research/Education Center for Excellence in Molecular Sciences, National Centre for Mass Spectrometry in Beijing, Institute of Chemistry Chinese Academy of Sciences (CAS), Beijing, P. R. China

These authors contributed equally: Yi-Fan Tian, Shuang-Jie Tan.

*email: ygguo@iccas.ac.cn

Supplementary Note 1

Specific description to the mechanical properties SEI components.

The "mechanical-property" used here measures the contribution of a SEI component on SEI structural stability confronting the evolving interface of Si anode during cycling, which can be specified to the resilience. The resilience (U_{max}) refers to the deformation energy within the whole elastic zone (that is, maximum elastic strain energy), which is proportional to elastic modulus (E) and fifth power of elastic strain limit (ε_Y)[1,2], defined by：

$$U_{max} = \frac{8}{15}\left(\frac{4}{5}\pi\right)^5 (1-\nu^2)^4 E \cdot {\varepsilon_Y}^5 \cdot r^3 \quad (1)$$

Herein, ν is the Poisson's ratio, r is radius of the indenter. High modulus LiF was a key SEI component facilitating stabilized interphase on the Si-based anodes.[3–5] Additionally, polymeric species including polycarbonates were also prevailing SEI components for their advantages in elastic strain limit.[6,7] These characteristics exhibit positive effect on the structural stability of SEI, which was regarded as the "favored" or "designed" SEI components in this work. In comparison, other components such as typical alkyl carbonates (like LEDC and LEC) and inorganic species (such as $Li_xPF_yO_z$) are considered to have "poor" mechanical property due to their lower resilience (lower modulus) compared to LiF and polycarbonates (Supplementary Table 1).[8–10]

Supplementary Note 2

Solubility of SEI in GBL solvent.

To confirm the dissolution of SEI in GBL solvent, an electrochemical method was applied here. In this method, Li||Si cells with modified electrolyte formulation were assembled and measured by a periodic lithiation-rest process. This lithiation-rest process starts with a galvanostatic cathodic step to form SEI on the silicon anode, followed by the 100-min rest without external current or potential polarization. In the ensuing 100-min rest process, the unstable SEI may dissolve and cease to passivate the electrode surface, which leads to the self-discharge phenomena. Notably, a high cut-off potential (0.2 V *vs.* Li^+/Li) was applied in the lithiation process to exclude the impact from the volume expansion on the stability of SEI. The formulation of the electrolytes used here did not contain FEC (see Experimental Methods, denoted as mGBL-based, mPC-based, mEC-based electrolytes for modified GBL-based, PC-based and EC-based electrolytes respectively), and $LiPF_6$ was also replaced by lithium bis((trifluoromethyl)sulfonyl)azanide (LiTFSI) to minimize the passivation effect from LiF in this experiment. As every single period shown in Supplementary Fig. 4, the potential evolution recorded in the rest process first experienced a rapid rise and then leveled off in mGBL-based electrolytes, indicating that the SEI was dissolved and failed to shield the anode from the electrolytes. Furthermore, the inter-period comparison demonstrates almost nothing stable on the electrode surface since the final voltage was as high as ~1.2 V. In contrast, the SEI in mEC-based electrolytes shows much lower stable potential that continually decreases cycle by cycle, indicating the accumulation of SEI species. mPC-based electrolytes present a similar potential evolution process to mEC-based electrolytes owing to the deposition of other reduction products despite the soluble LPDC. Remarkably, the potential was finally stabilized to nearly 0.4 V once the F source was introduced in GBL-based electrolytes, indicating the suppressed self-discharge situation and stabilized SEI according to the yellow curve in Supplementary Fig. 4. Such dramatic contrasts verify the solubility of GBL towards most conventional SEI components as well as the patching effect of the F-contained components, which make it possible to screen and modulate specific SEI components among the complex reduction products of electrolytes.

Supplementary Note 3

Practicability of solvent-assisted optimization strategy.

Considering the flooded electrolyte conditions in coin cells, it remains to be ambiguous whether this strategy involves negative effects in widely applied cell formats with limited electrolytes amount, as the dissolved species are concentrated in lean electrolyte conditions and probably interfere with the bulk electrolyte and cathode. Therefore, we conducted extra experiments aiming at exploring the evolutions of electrolyte and cathode surface to elucidate the potential effect in the typical electrolyte to capacity ratio of 3g A^{-1} h^{-1}. In brief, 15 pieces of ~4 mA h cycled silicon electrodes were collected from the Li||Si half cells with the classic EC-based electrolytes. In the next, these electrodes were fully soaked in 180 mg GBL-based electrolytes for 72 h, followed by Raman spectroscopy to analyze its discrepancy from the pure GBL solvent and fresh GBL-based electrolytes. According to the Raman spectra in Supplementary Fig. 24, the GBL-based electrolytes exhibit highly consistent with the fresh electrolyte without obvious peak shift or other evolution, indicating intact solvent structure and state. In regard to the cathodes, the XPS was carried out to identify the components on cathode electrode interphase (CEI), as shown in Supplementary Fig. 25. Based on the O 1s and C 1s spectra, the CEI formed in GBL-based electrolytes exhibit similar composition and relative amount to that of PC and EC-based electrolytes, while LiF was found only in F 1s spectra of GBL and PC-based electrolytes. Notably, the LiF was regarded as a critical component capable of effectively inhibiting the dissolution of transition metal and stabilizing the cathode structure, hence it can be concluded that the GBL-based electrolytes exert a positive effect on the cathodes.

Supplementary Note 4

Analysis of the force-displacement curves.

The nanoindentation test was also conducted to uncover the SEI evolution in response to the stress, which provides detailed predictions for the evolution of the SEI under volume variation of the electrode. During the test, the indenter with load (>50 nN) was applied to intentionally impale the SEI and then retract (Supplementary Fig. 30), and the corresponding force-displacement curves were recorded, as shown in Fig. 4d-f. According to the indenter state and the strain of the measured SEI, the obtained force-displacement curve can be divided into several segments, and the mechanical properties of the SEI during each segment are discussed below. The red curve in Fig. 4d recorded the strain evolution of SD-SEI in response to the indenter gradually approaching and impaling it. Specifically, point (α) represents the time when the indenter started to contact the SD-SEI surface, followed by an elastic deformation region of SD-SEI until point (β). In the following, plastic deformation happens from point (β) to point (γ), and then the indenter directly contacts the silicon electrodes after point (γ), which means the distance between the point (α) and (γ), t, equals to the thickness of SEI. The position of point (β) was precisely located as the first deviation point from the fitting curve of Hertz's contact model between point (α) and (y) (Supplementary Fig. 31). Specifically, in the Hertz contact model, the relationship between applied force (F) and the indentation (δ) in the elastic deformation region of SEI can be described as followed:

$$F = \frac{4}{3}\frac{E}{1-\nu^2}\sqrt{r}\delta^{3/2} \qquad (2)$$

where E is the Young's modulus, ν is the Poisson's ratio, r is radius of the indenter.

Similarly, the point (γ) was determined as the first deviation point from the fitting curve of Hertz's contact model starting from point (γ). In pursuit of an appropriate indicator to evaluate the tolerance of SEI towards volume expansion, we focus on the elastic strain limit: the distance between the point (α) and (β), which could demonstrate the maximum deformation under the premise of maintaining the SEI reversibility.

The validity of the force-displacement curves was ensured by the following points: (1) The data were collected in the relatively flat area, so nearly no torque was generated during the measurement. (2) The electrode, together with the attached micron-sized Si particles was

compacted during the cell assembly and the ensuing volume expansion process. Therefore, the pressure applied during the measurement is less likely to cause structural evolution, which provided a relatively stable environment for the measurement. (3) The sharp contrast between Si and SEI components (>150 GPa *vs.* ~1 GPa) further enhanced the accuracy of the measurement.

The indentation experiment on an acrylic plate further valid the result from the force-displacement curves. According to the result, the Young's modulus of the acrylic plate was 2.2 GPa (Supplementary Fig. 32), which agrees with result in the previous report (2.4 GPa[11]).

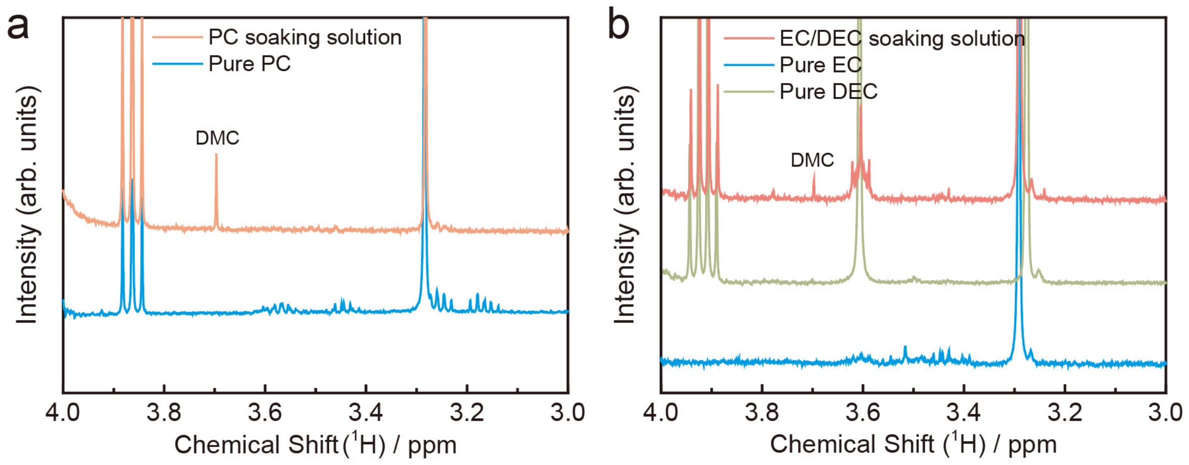

Supplementary Fig. 1. NMR spectra of pure PC and PC solvent after soaking the cycled Si electrodes (a), pure EC/DEC mixed solvent and EC/DEC mixed solvent after soaking the cycled Si electrodes (b).

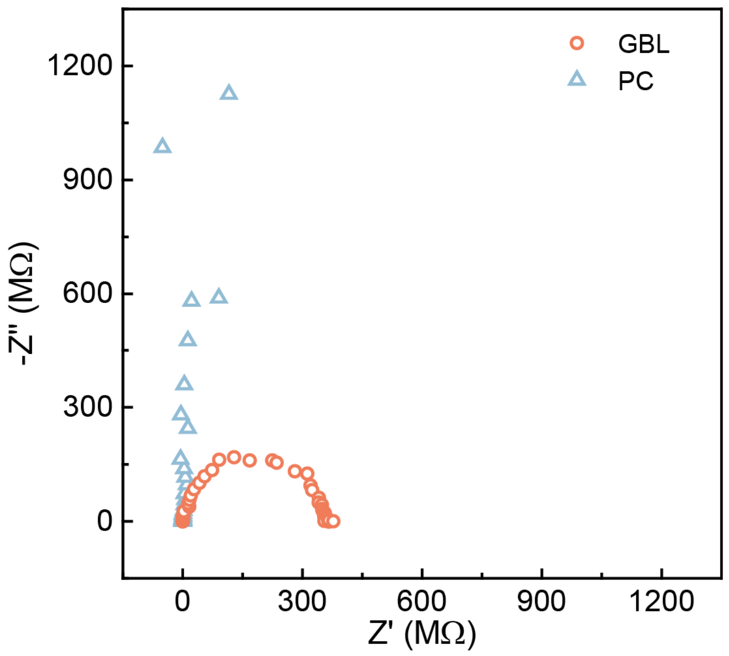

Supplementary Fig. 2. Nyquist plots of stainless steel (SS)||SS coin cell with various soaking solvents.

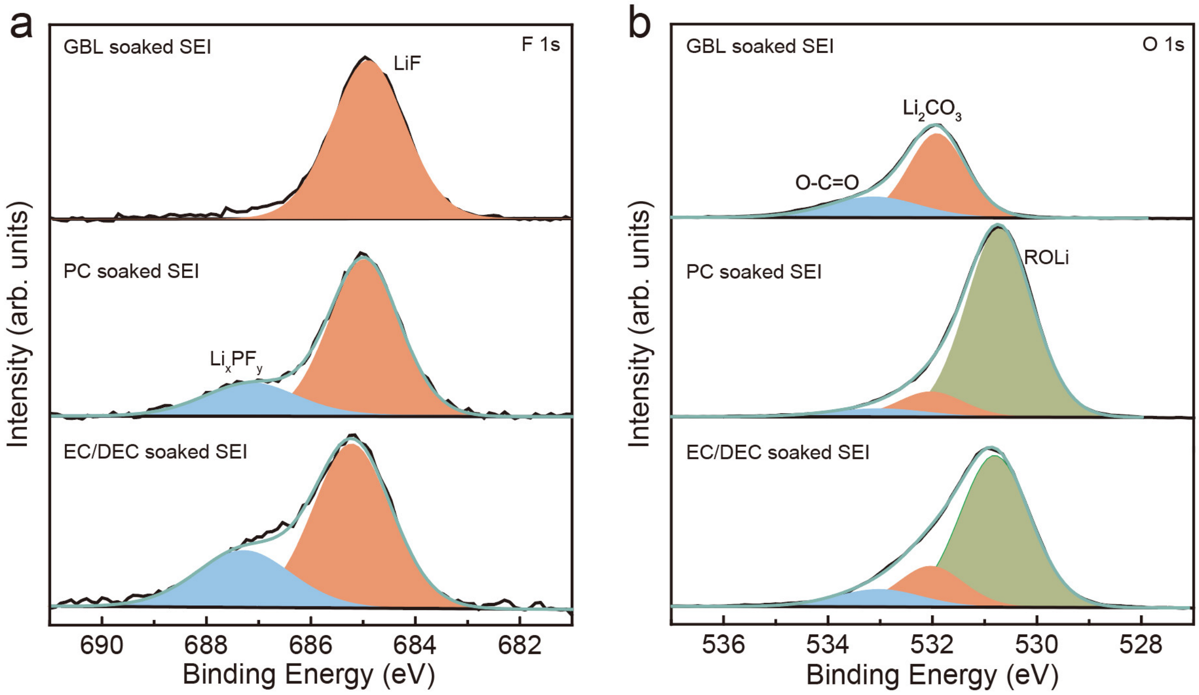

Supplementary Fig. 3. The XPS spectra characterization of cycled silicon electrode after soaked with various solvent: F 1s (a), O 1s spectra (b).

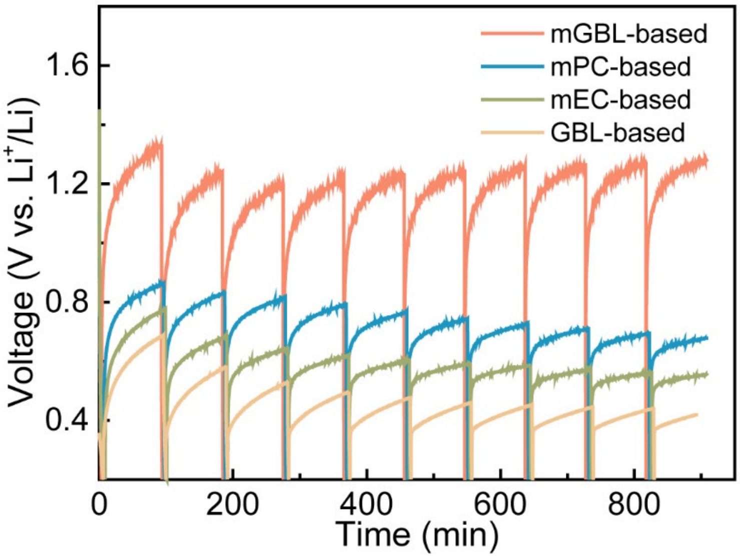

Supplementary Fig. 4. Voltage profiles of silicon anodes with galvanostatic cathodic step followed by 100-min rest step in one period. During the cathodic step, the cut-off potential was set to be 0.2 V with current density of 0.1C. 1C=3000 mA g^{-1}.

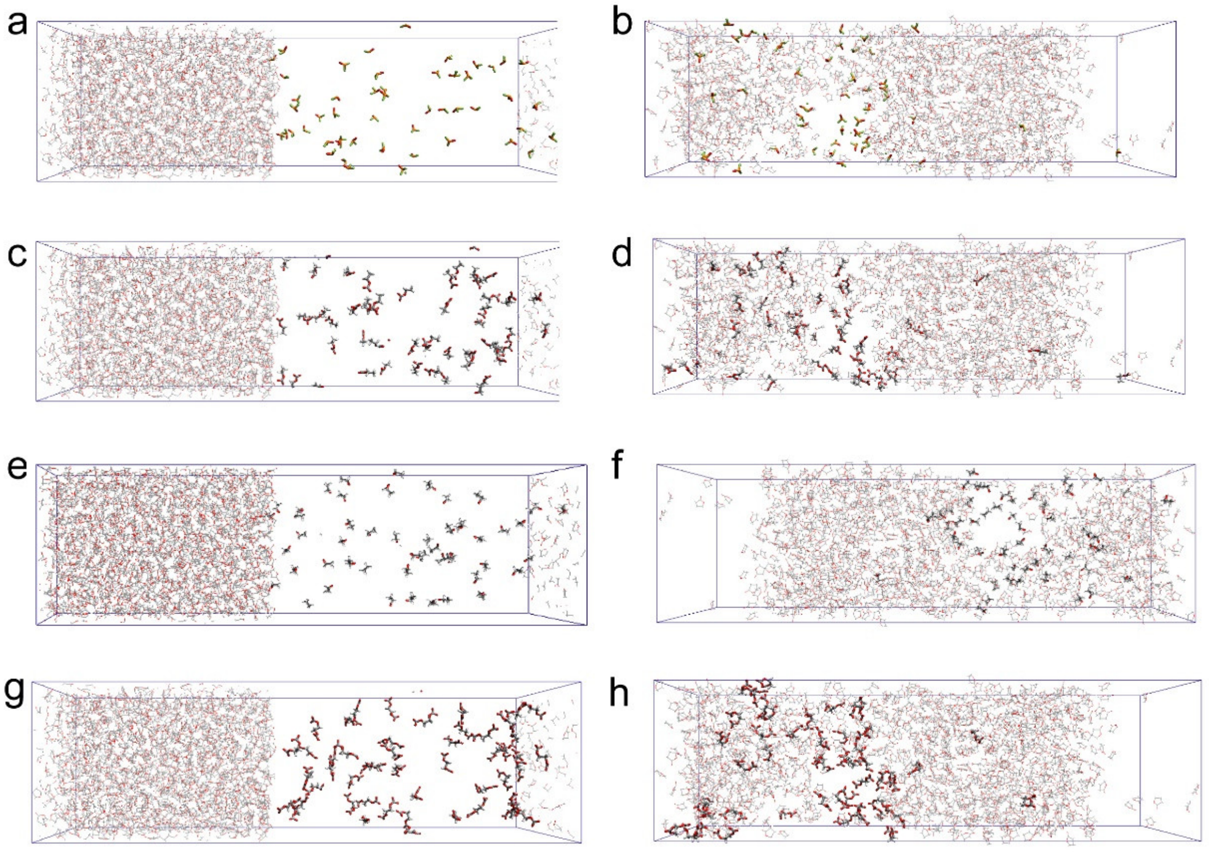

Supplementary Fig. 5. Snapshots of the MD simulaitions of PF_2O^- (a-b), $CH_3CH_2CO_3^-$ (c-d), $CH_3CH_2O^-$ (e-f) and $(CO_3)CH_2CH_2CO_3^{2-}$ (g-h) with GBL molecules at the beginning (a, c, e, g) and after 150 ns relaxation process (b, d, f, h).

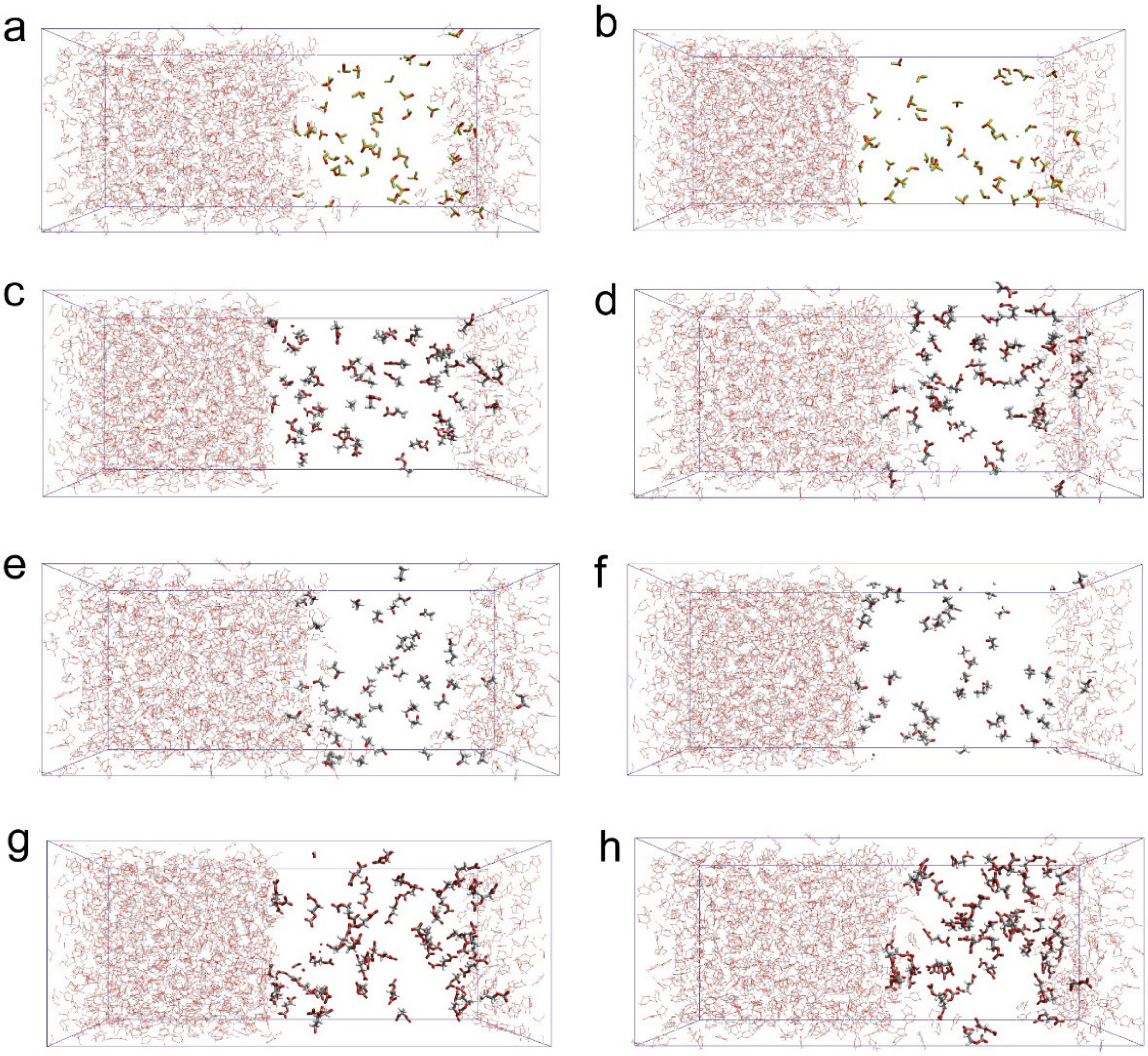

Supplementary Fig. 6. Snapshots of the MD simulaitions of PF_2O^- (a-b), $CH_3CH_2CO_3^-$ (c-d), $CH_3CH_2O^-$ (e-f) and $(CO_3)CH_2CH_2CO_3^{2-}$ (g-h) with EC molecules at the beginning (a, c, e, g) and after 150 ns relaxation process (b, d, f, h).

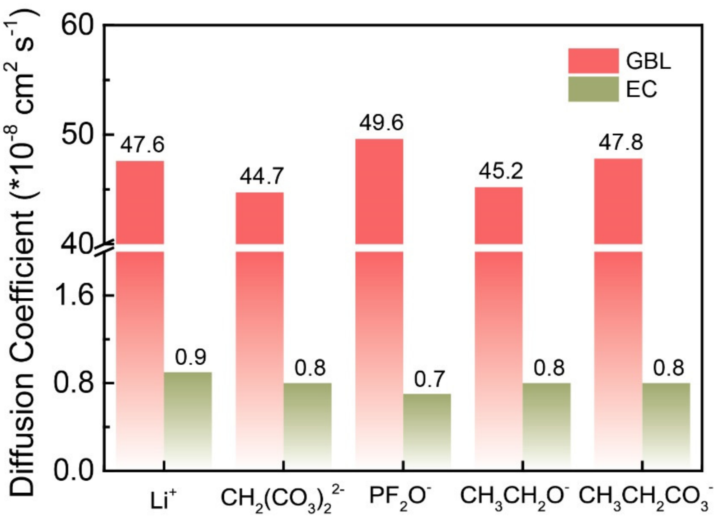

Supplementary Fig. 7. The calculated diffusion coefficient of selected SEI species in different solvents based on the mean square displacement results in Fig. 1h.

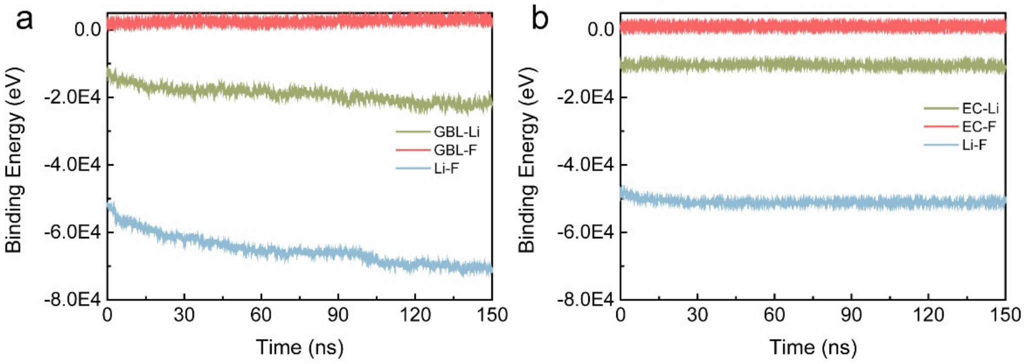

Supplementary Fig. 8. Binding energy of solvent-Li, solvent-F and Li-F bond in GBL (a) and EC (b) solvents throughout the 150 ns simulations period.

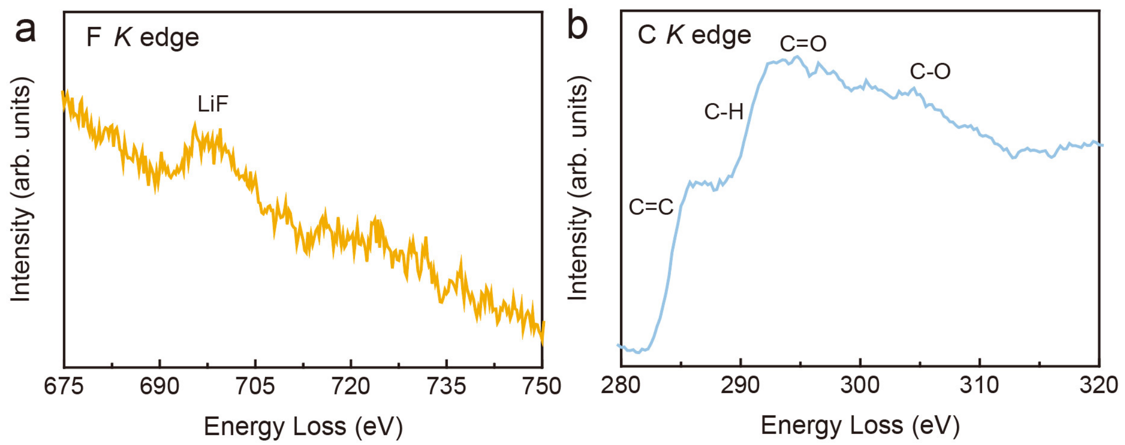

Supplementary Fig. 9. Typical F K-edge (a) and C K-edge (b) EEL spectra of SD-SEI.

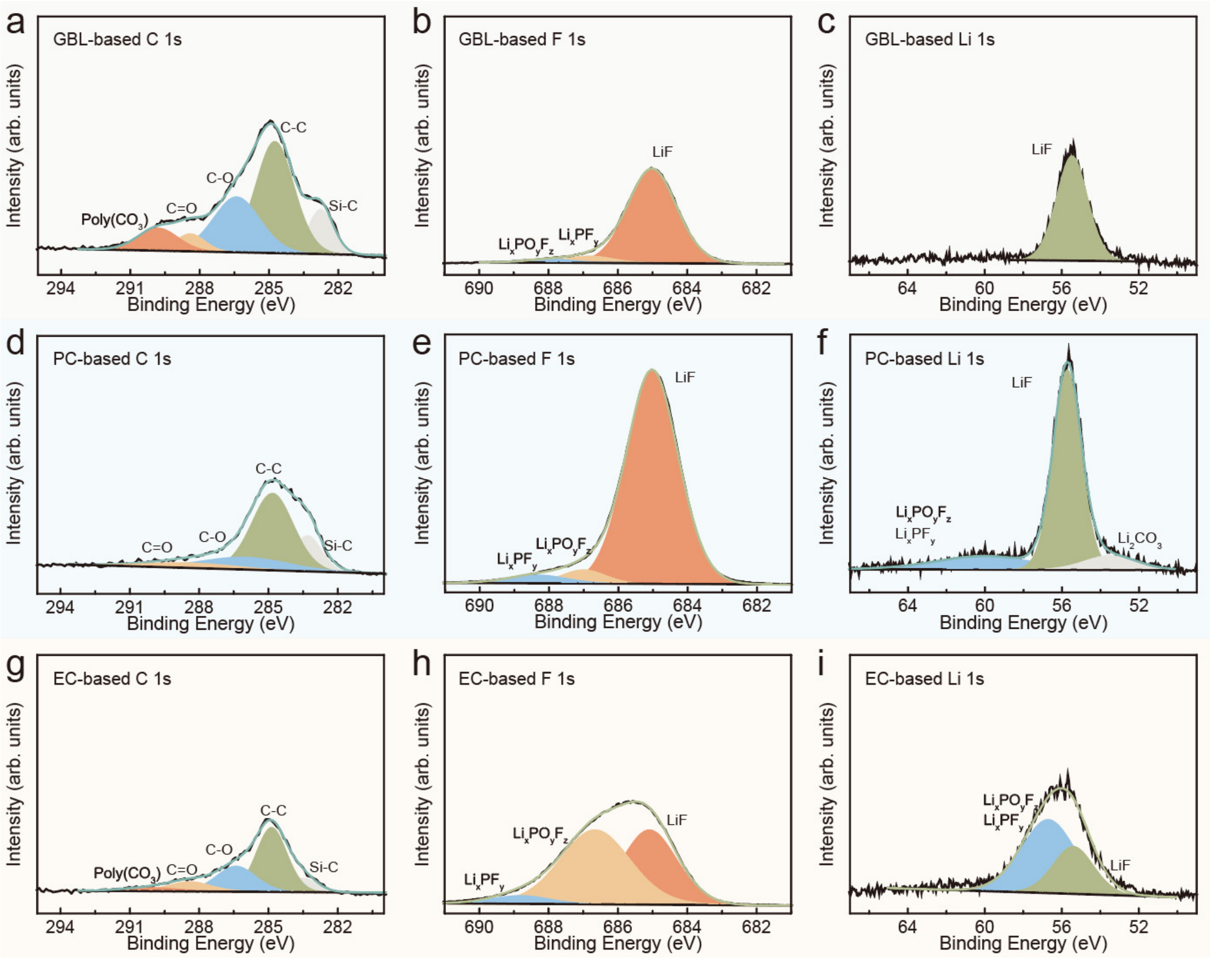

Supplementary Fig. 10. XPS spectra of a-c, SD-SEI: C 1s (a), F 1s (b), Li 1s (c); d-f, F-SEI: C 1s (d), F 1s (e), Li 1s (f); and g-i, c-SEI: C 1s (g), F 1s (h), Li 1s (i).

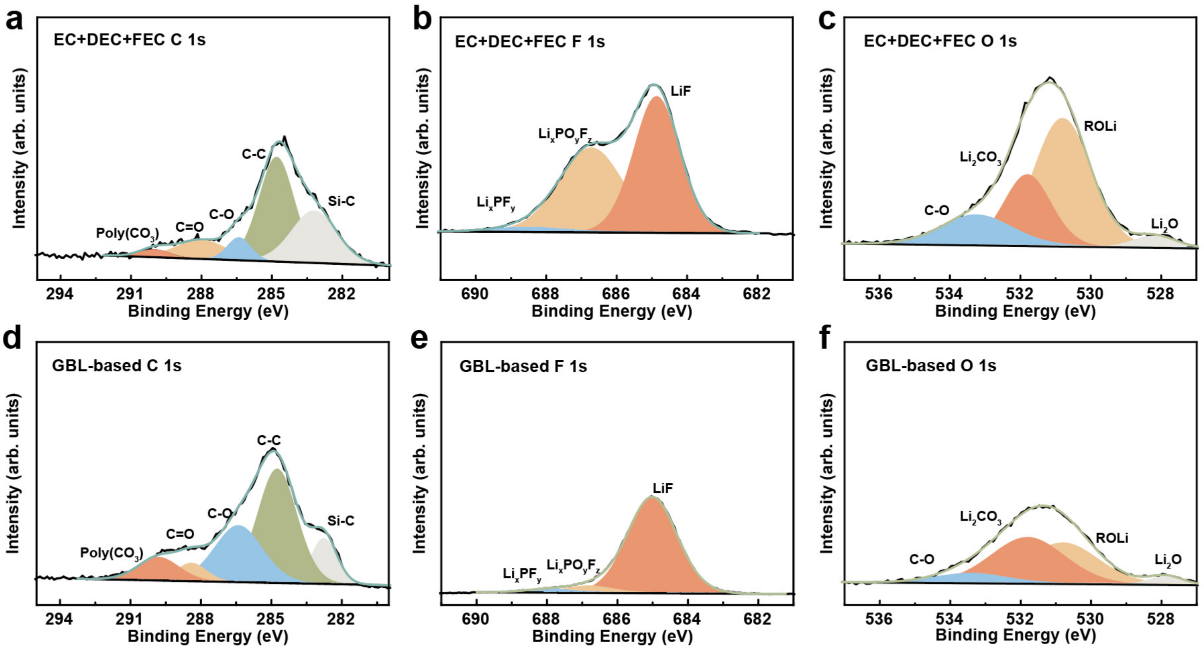

Supplementary Fig. 11. XPS characterizations of the SEI on the micron-sized Si anode in (a-c) the EC-FEC electrolyte and the (d-f) GBL-based electrolyte after 20 cycles.

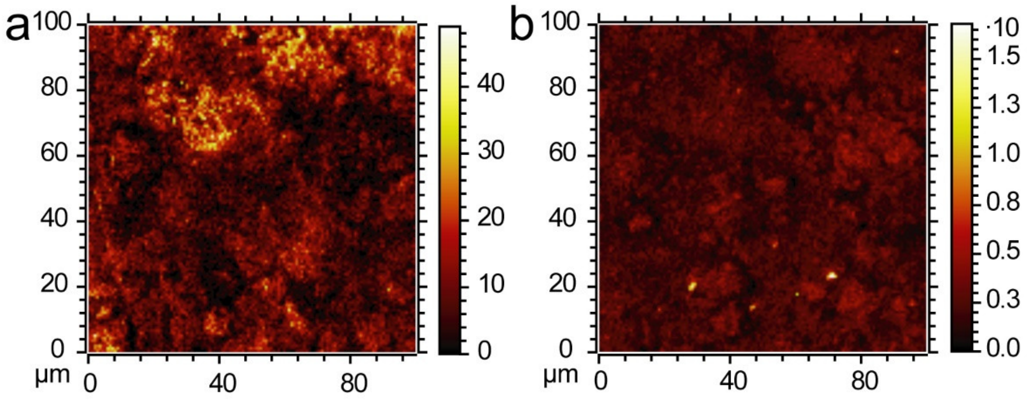

Supplementary Fig. 12. ToF-SIMS secondary ion images of the cycled micron-sized silicon electrodes of LiF_2^- (a) and O^- (b) on cycled electrodes in PC-based electrolytes. The studied electrodes have performed 20 cycles before carefully extracted and cleaned with DMC solvent.

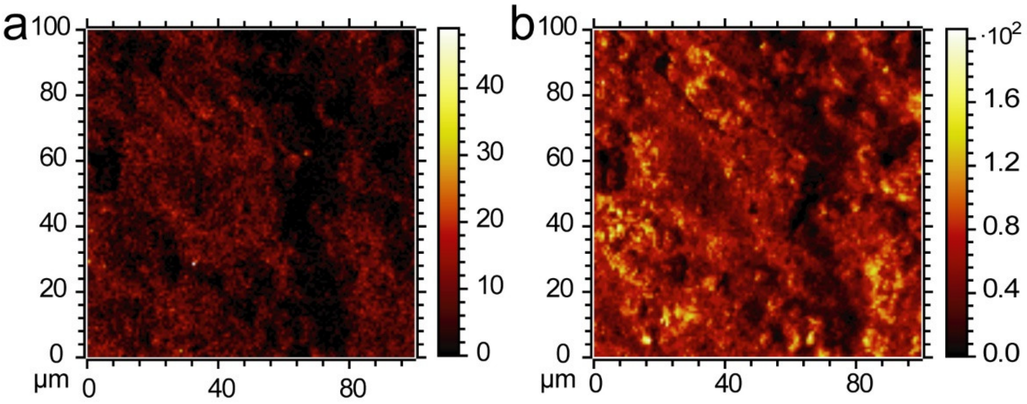

Supplementary Fig. 13. ToF-SIMS secondary ion images of the cycled micron-sized silicon electrodes of LiF_2^- (a) and O^- (b) on cycled electrodes in EC-based electrolytes. The studied electrodes have performed 20 cycles before carefully extracted and cleaned with DMC solvent.

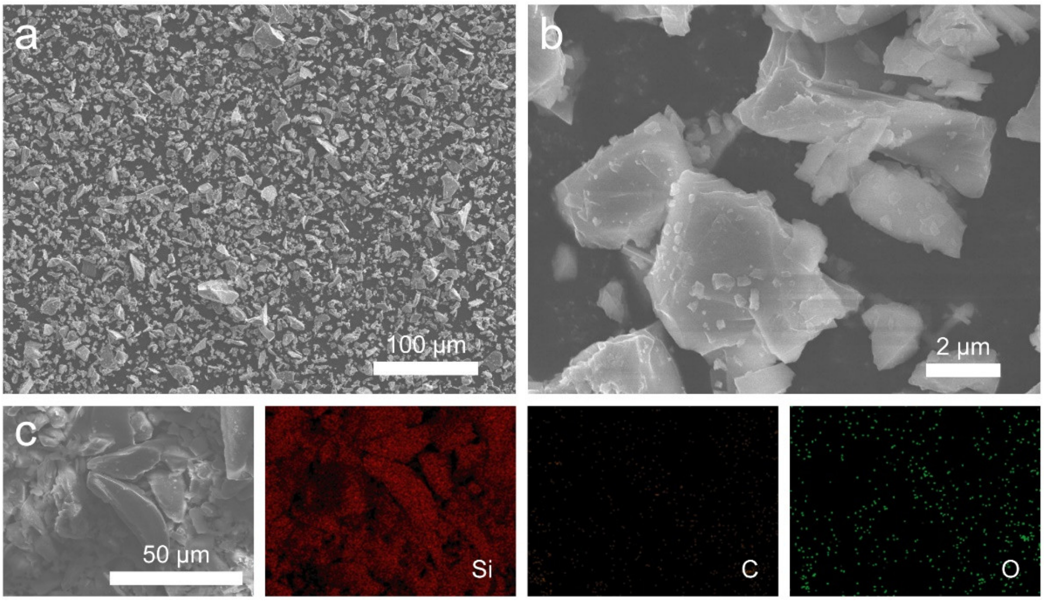

Supplementary Fig. 14. Typical SEM images of micron-sized silicon (a-b) and corresponding elemental mapping images (c). The silicon particles exhibit irragular shape with various sizes, and the carbon elemental mapping images prove the absence of carbon layer.

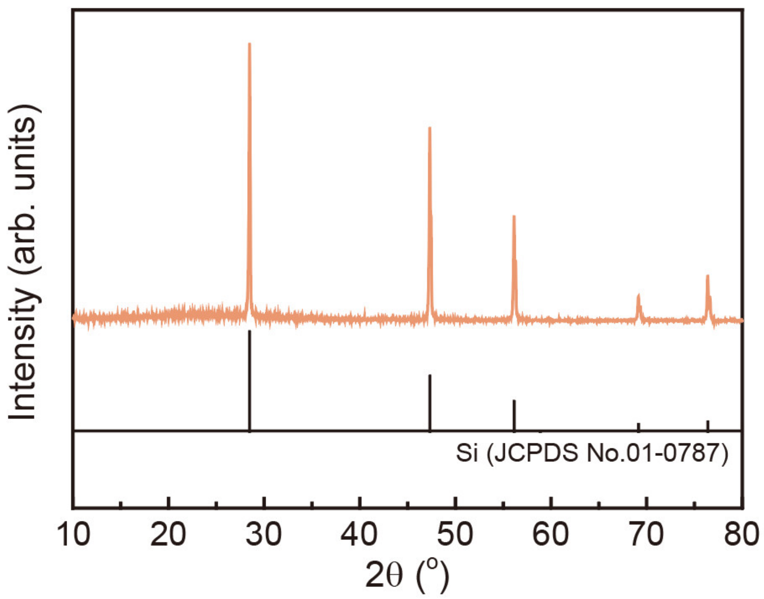

Supplementary Fig. 15. XRD patterns of micron-sized silicon. The diffraction peaks were rather sharp with narrow half-peak width, which can be indexed to crystal Si (JCPDS No.01-0787).

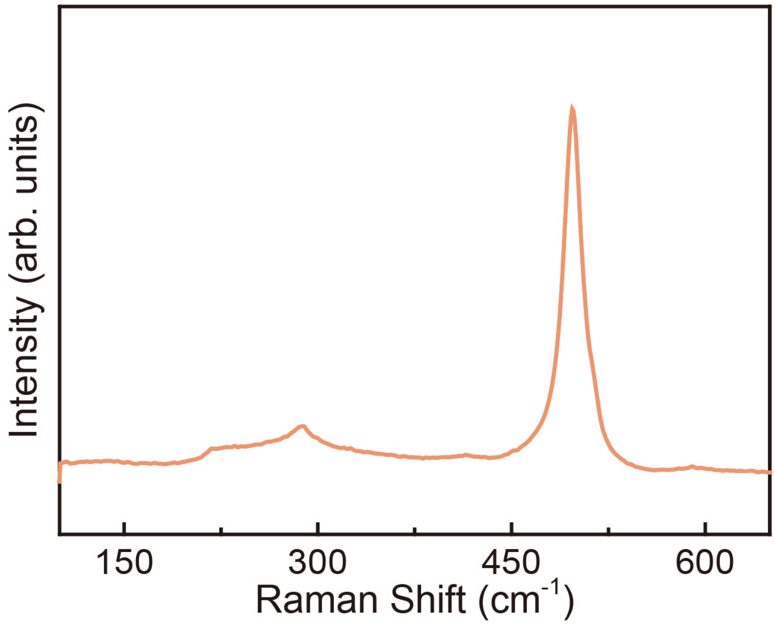

Supplementary Fig. 16. Raman spetra of micron-sized silicon. The sharp peak located at ~520 cm^{-1} are generated by optical phonon modes of c-Si, indicating the perfect crystallinity in this micron-sized silicon.

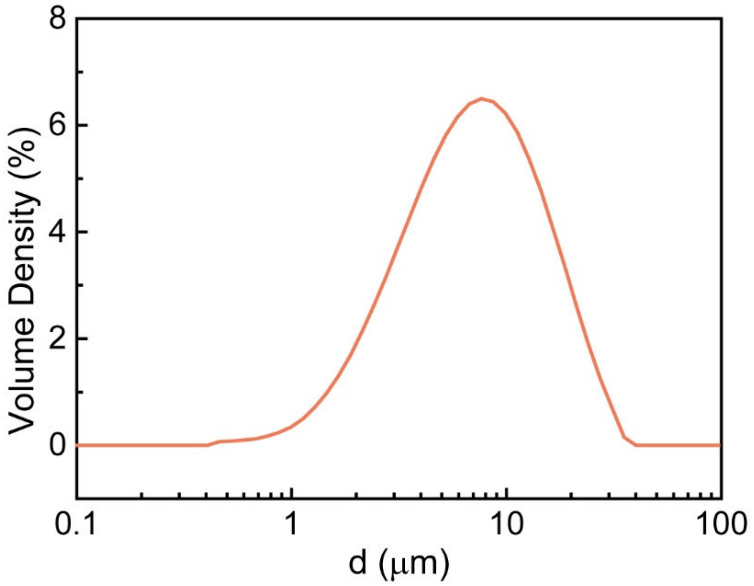

Supplementary Fig. 17. Particle distribution function of micron-sized silicon. According to the curves, the medium diameter was measured to be 7.8 μm with wide size distribution, which meets the findings of morphology in Supplementary Fig. 14. Such configuration of material structure and size would lead to aggressive volume expansion problems during lithiation process, but approximately in line with the practical LIBs anode materials and capable of examining the mechanical properties of SEI.

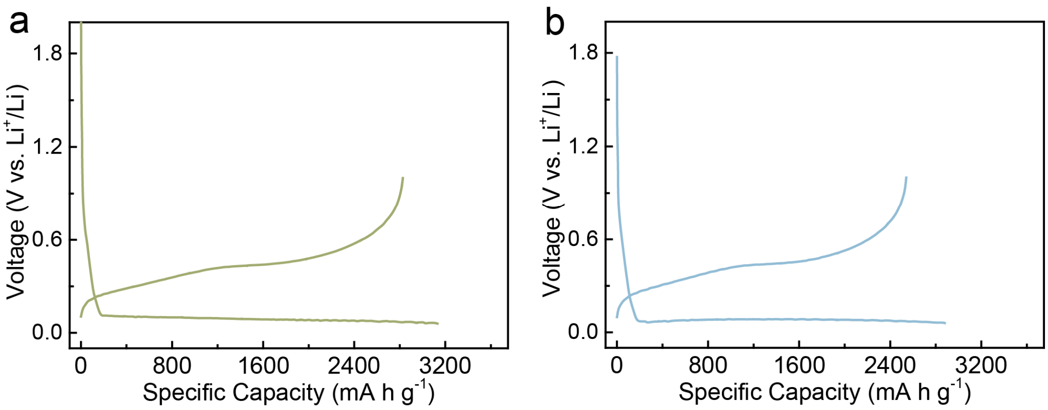

Supplementary Fig. 18. Typical charge/discharge profiles from 0.06 V to 1.0 V at 0.05C (1C=3000 mA g^{-1}) in PC (a) and EC-based (b) electrolytes, respectively.

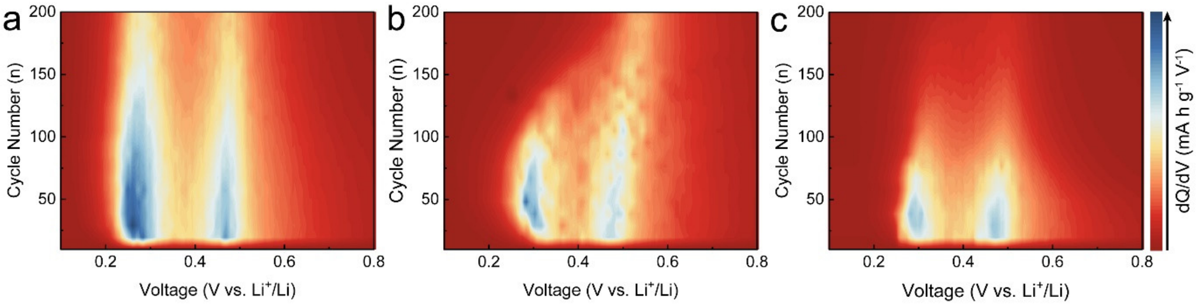

Supplementary Fig. 19. Contour map of differential capacity (dQ/dV) versus voltage in different cycling numbers in GBL-based electrolytes (a), PC-based electrolytes (b) and EC-based electrolytes (c). At the beginning of the cycling measurement, all the systems show two dominant peaks at around 0.3 V and 0.5 V relating to the conversion between Li_xSi and Si, inferring an identical (de)lithiation process mechanism using these electrolytes. During cycling, the peak intensity declined remarkably for Si anodes in both PC and EC-based electrolytes. Besides, discernible peak shift indicates the increasing polarizations in PC-based electrolytes, which accounts for the high average Coulombic efficiency while low capacity retention. These above effects seem to be negligible for GBL-based electrolytes, implying better cycling stability and stable interfacial issues compared to the above control samples.

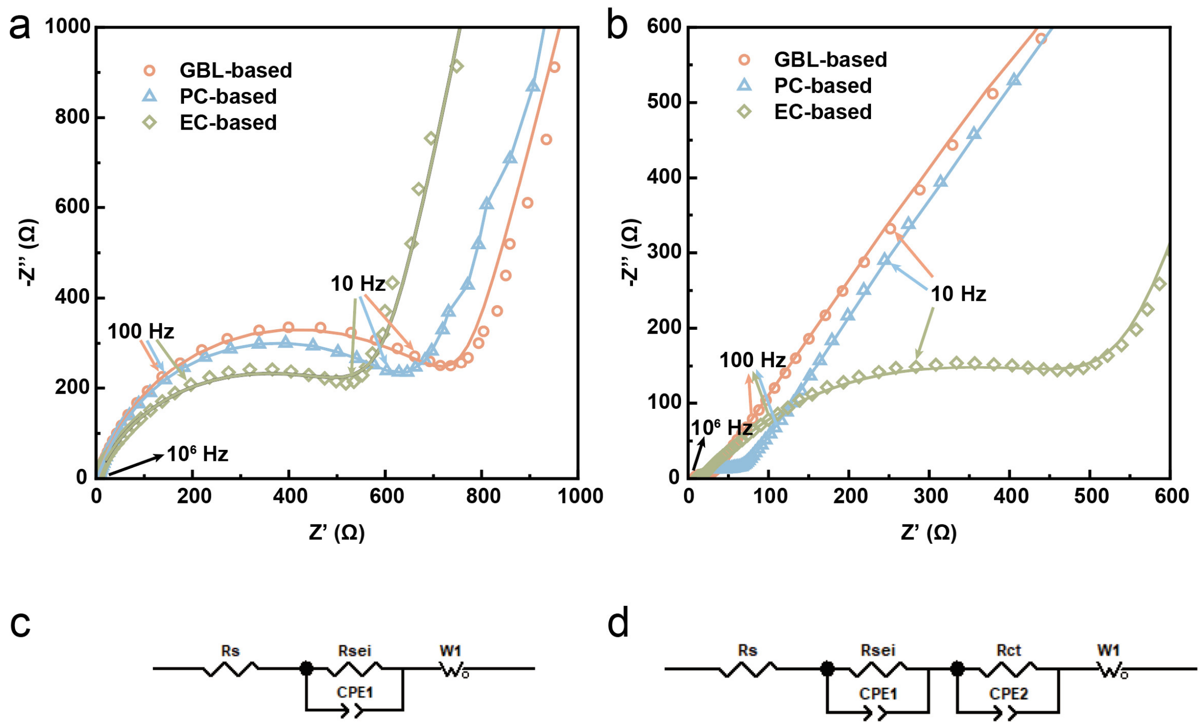

Supplementary Fig. 20. Nyquist plots of Li||Si half-cell with GBL-based electrolyte, PC-based electrolyte and EC-based electrolyte in pristine state (a) and after 200 cycles (b). The corresponding equivalent circuit applied in pristine state (c) and cycled state (d). R_s, R_{SEI} and R_{ct} represent the solution resistance, SEI resistance and charge transfer resistance, respectively. CPE, CPE1 and CPE2 stands for the relative double-layer capacitance. Ws represents the Warburg impedance related to lithium-ions diffusing. The semi-cycles located at higher frequency indicates the R_{SEI}, while the other one at lower frequency represents the R_{ct}. The fitting results were plotted as the solid curves in (a) and (b). In terms of R_{SEI}, the SD-SEI shows lower value compared with that of F-SEI and c-SEI (1.6 Ω *vs.* 67.6 Ω *vs.* 193.5 Ω for SD-SEI, F-SEI and c-SEI, see Supplementary Table 2), indicating the favored kinetics properties and excellent stability of SD-SEI.

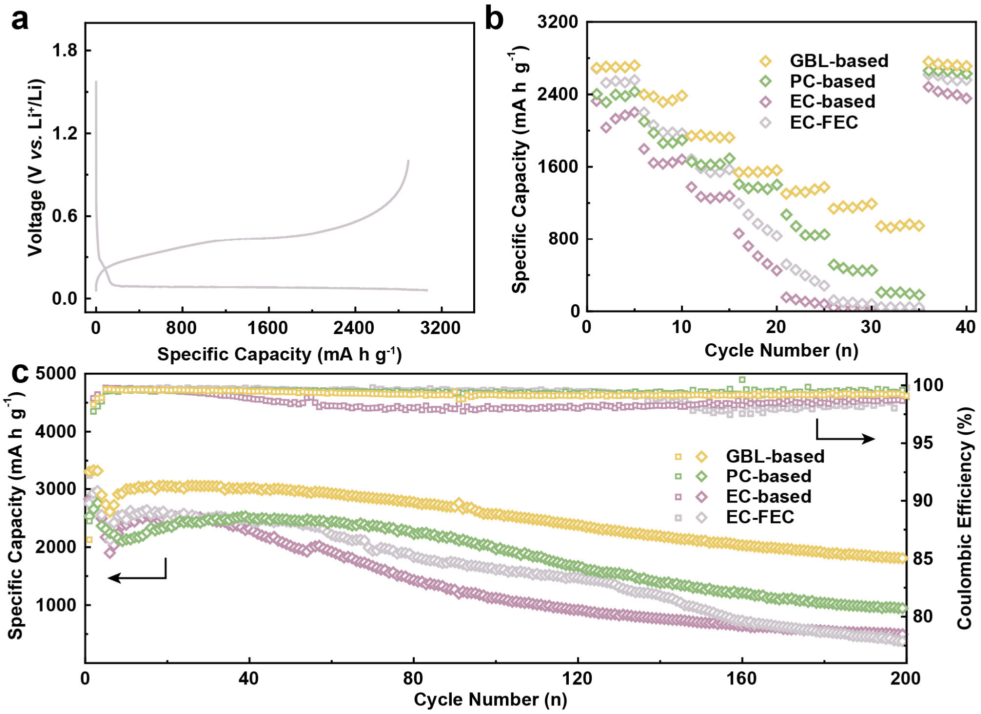

Supplementary Fig. 21. a, Typical charge/discharge profiles of the micron-sized Si anode in the EC-FEC electrolyte from 0.06 V to 1.0 V at 0.05C. b, Rate performance comparison at current density of 0.2C, 0.5C, 1C, 2C, 3C, 4C and 5C. c, Cycling performance and CEs of the micron-sized Si anode in various electrolytes at 0.2C. 1C=3000 mA g^{-1}.

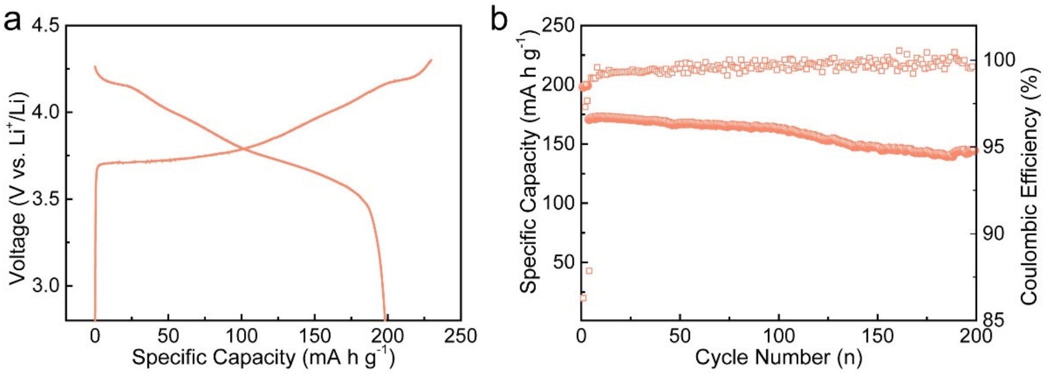

Supplementary Fig. 22. Typical charge/discharge profiles (a) from 2.8 V to 4.3 V at 0.1C and cycling performance at 0.5C (b) of NCM811||Li half-cell cycled in GBL-based electrolyte, where 1C=180 mA g^{-1}.

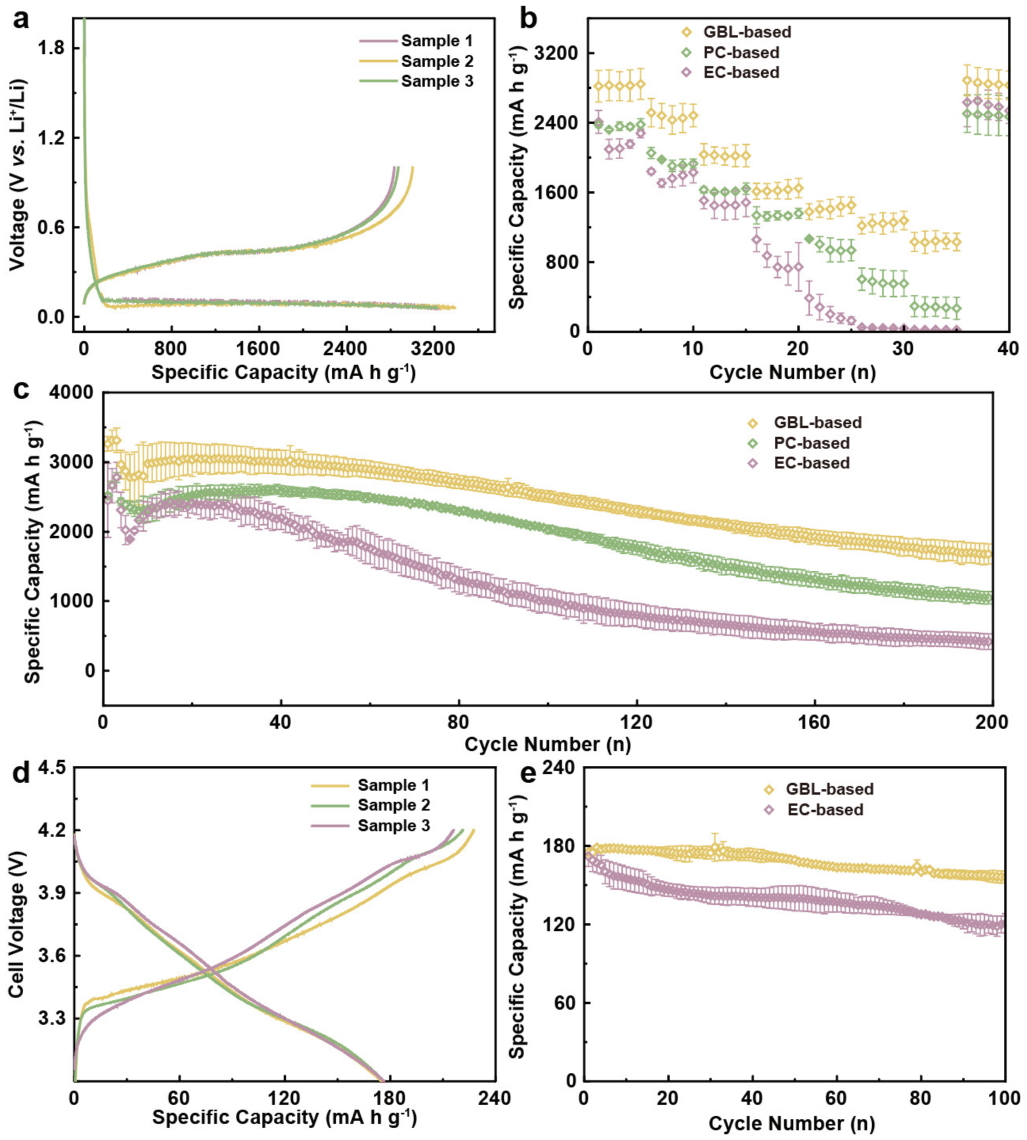

Supplementary Fig. 23. a, Typical charge/discharge profile of the micron-sized Si anode in the GBL-based electrolyte from 0.06 V to 1.0 V at 0.05C. b, Rate performance comparison at current density of 0.2C, 0.5C, 1C, 2C, 3C, 4C and 5C. c, Cycling performance and CEs of the micron-sized Si anode in various electrolytes at 0.2C. 1C=3000 mA g^{-1}. d-e, Typical charge/discharge profiles from 3.0 V to 4.3 V (d) and cycling performance (e) of the Si||NCM811 full-cells using various electrolytes at 0.5C, where 1C=180 mA g^{-1}. The error bars are plotted based on the mean of the results from the parallel tests and the corresponding standard deviations.

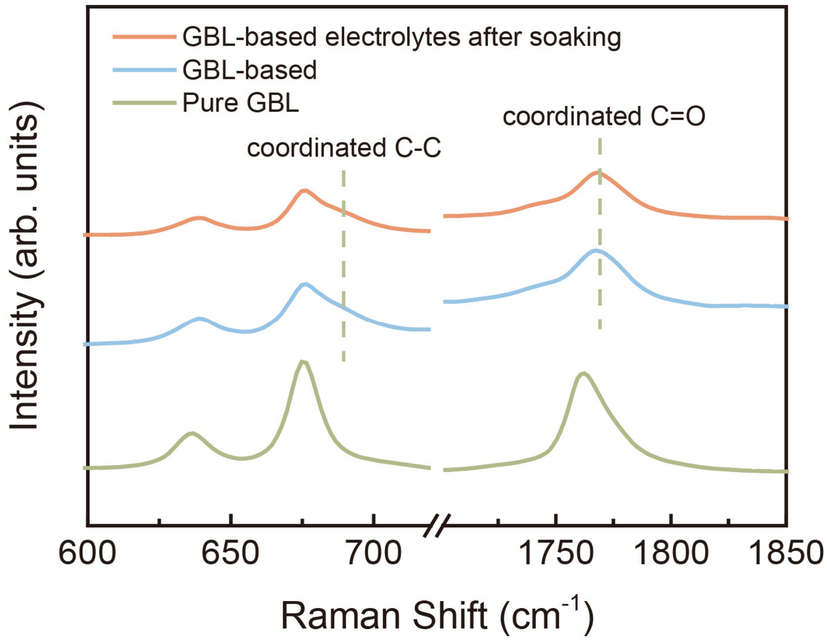

Supplementary Fig. 24. Raman spectra of pure GBL, GBL-based electrolyte and GBL-based electrolyte after soaking the cycled silicon electrodes. The studied electrodes have performed 20 cycles before soaked in corresponding electrolytes.

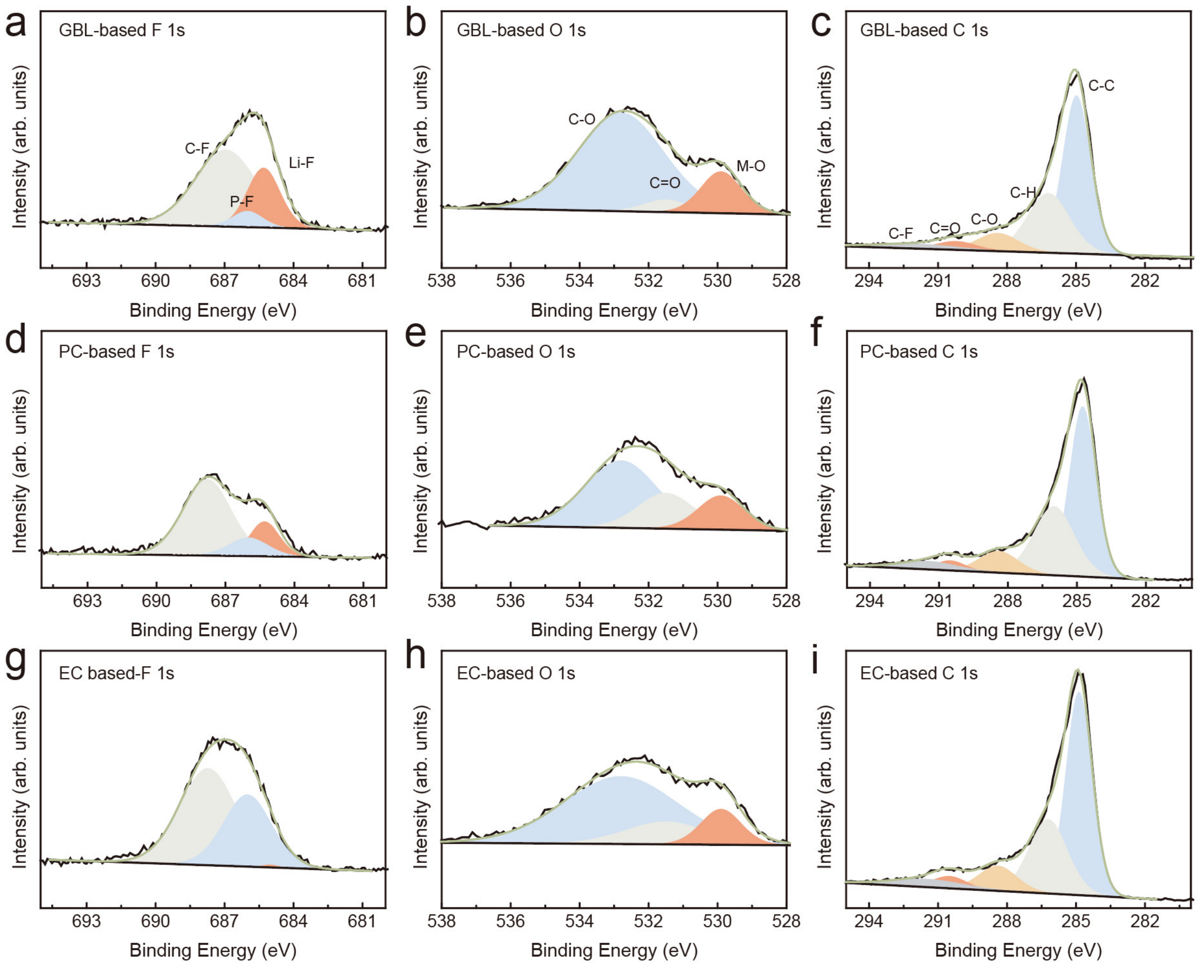

Supplementary Fig. 25. XPS characterization of the CEI in NCM811 electrodes cycled in various electrolytes. The studied electrodes have performed 20 cycles in corresponding electrodes before carefully extracted and cleaned with DMC solvent.

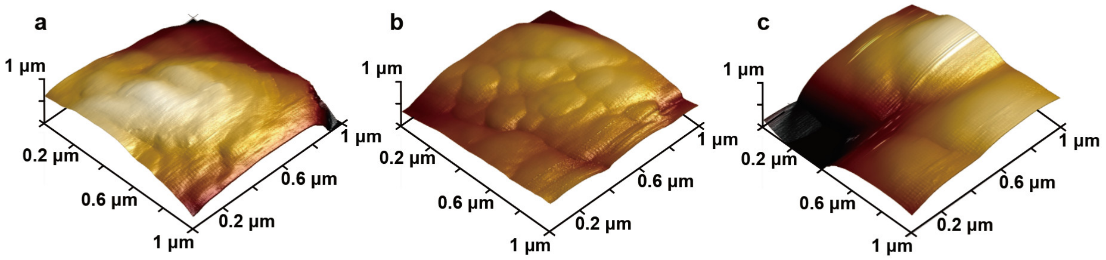

Supplementary Fig. 26. 3D morphology images of cycled micron-sized Si anode with (a) SD-SEI, (b) F-SEI and (c) c-SEI based on AFM. The studied electrodes have performed 20 cycles in corresponding electrode before carefully extracted and cleaned with DMC solvent.

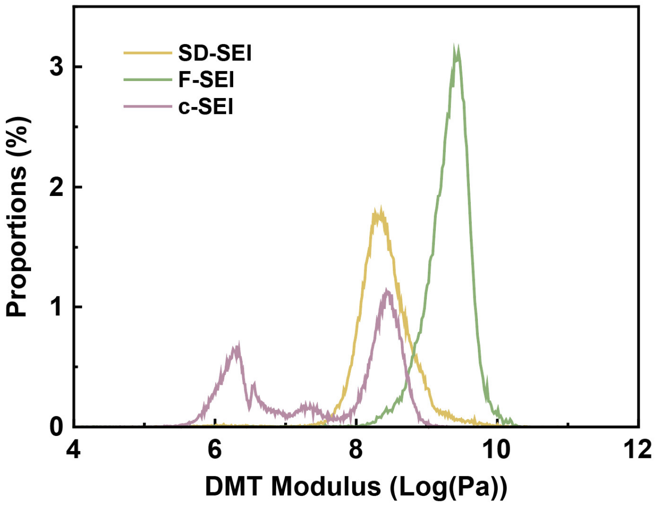

Supplementary Fig. 27. Modulus distribution of various SEIs.

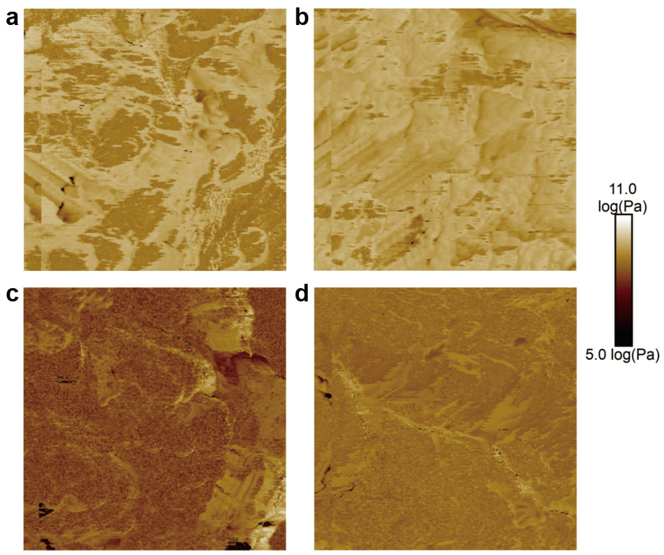

Supplementary Fig. 28. Derjaguin−Muller−Toporov (DMT) modulus mappings of SD-SEI of the Si anodes after (a) 1st, (b) 10th, (c) 20th and (d) 50th cycle.

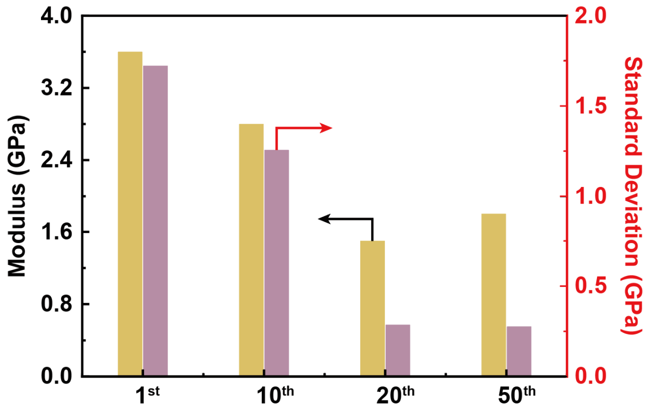

Supplementary Fig. 29. Comparison of modulus and corresponding standard deviation of SD-SEI after different cycling numbers.

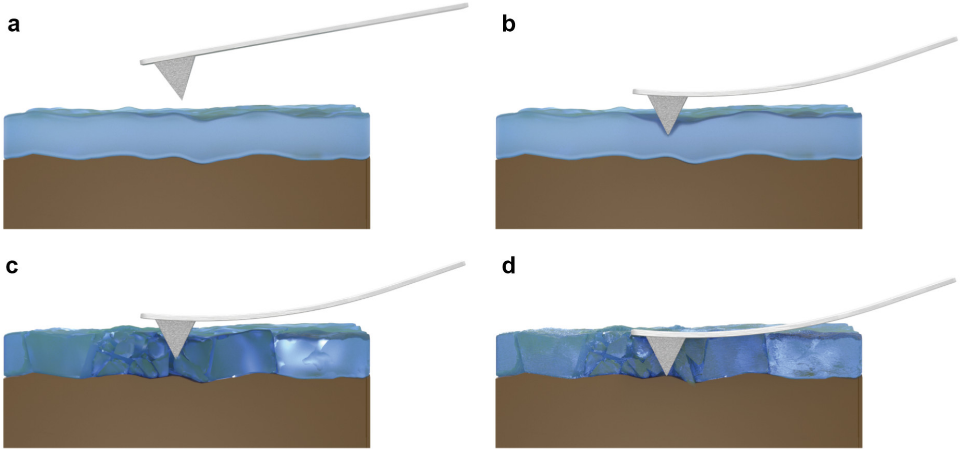

Supplementary Fig. 30. Schematic diagram of the force-displacement curve test process based on AFM, including (a) no contact, (b) elastic deformation, (c) plastic deformation of SEI, (d) penetrating the SEI layer and contacting the Si anode surface.

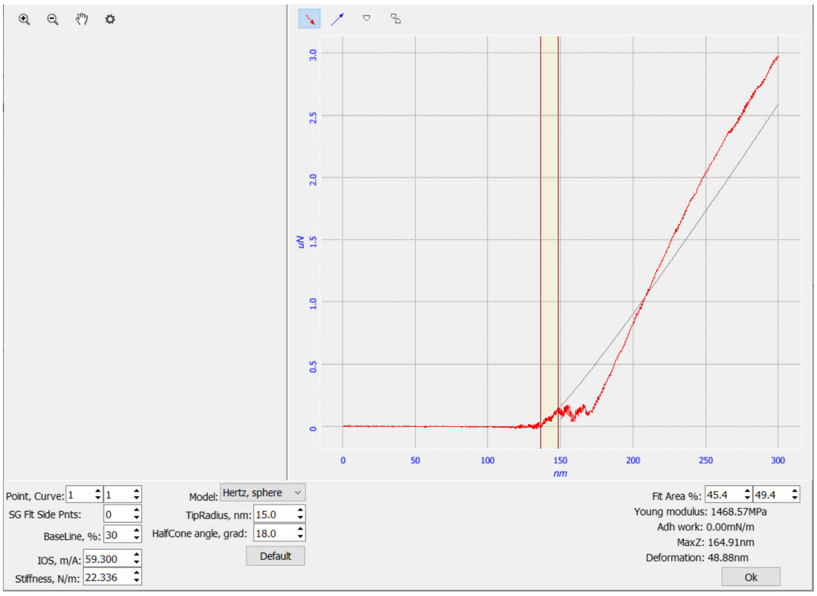

Supplementary Fig. 31. Fitting process of force-displacement curve.

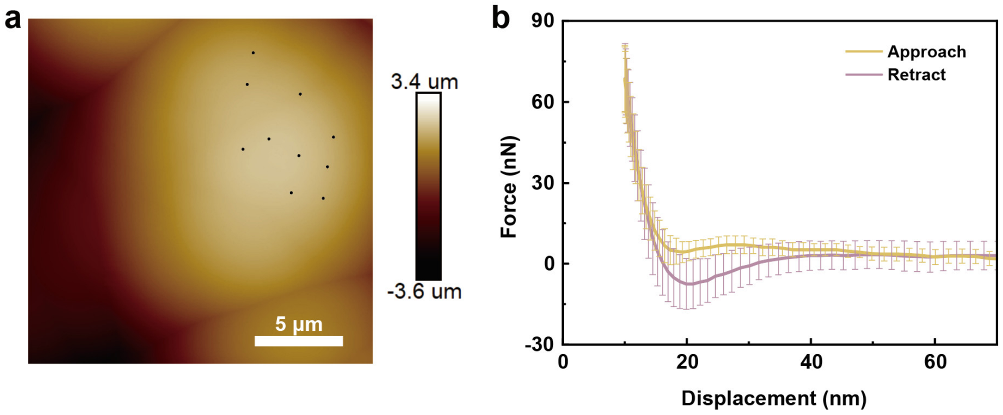

Supplementary Fig. 32. a, Morphology of acrylic plate. b, Force-displacement curve on an acrylic plate. The error bars are plotted based on the mean of the results from selected points in Supplementary Fig.32a (the black points) and the corresponding standard deviations.

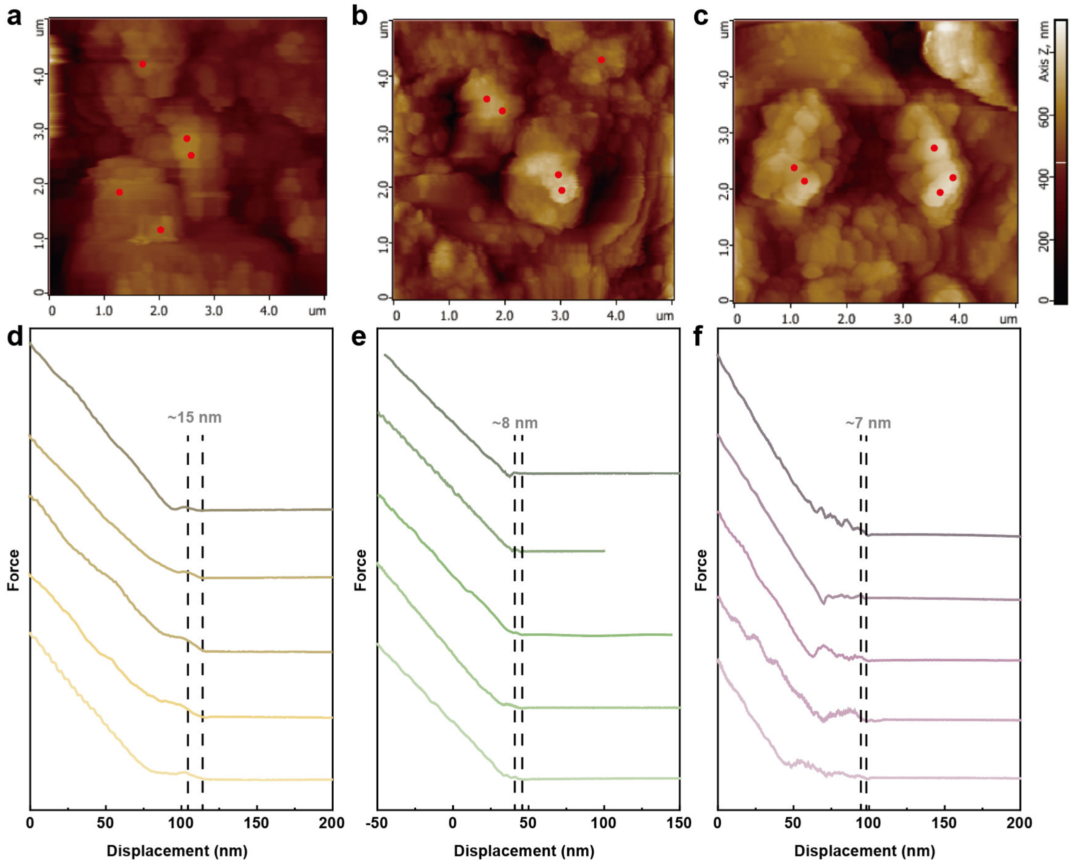

Supplementary Fig. 33. a-c. 2D morphology images of (a) SD-SEI, (b) F-SEI and (c) c-SEI based on AFM. d-f, force-displacement curves of (d) SD-SEI, (e) F-SEI and (f) c-SEI collected from the red points in the corresponding morphology images.

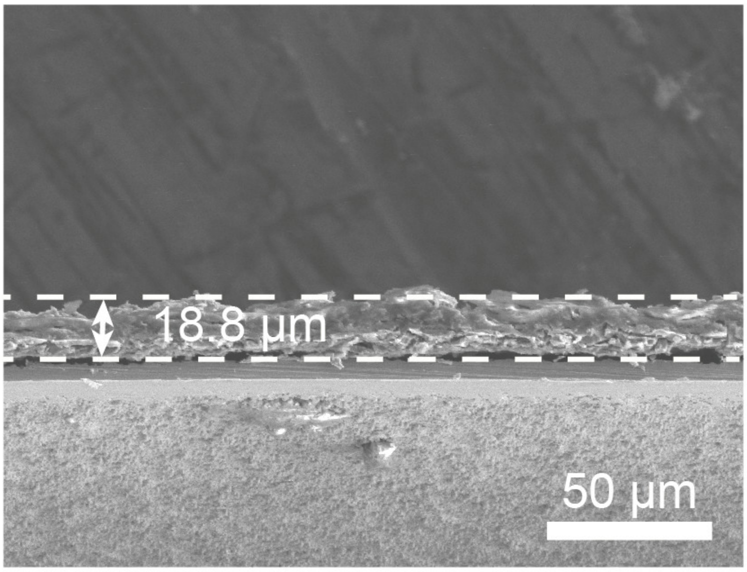

Supplementary Fig. 34. Typical cross-sectional SEM image of pristine silicon electrodes.

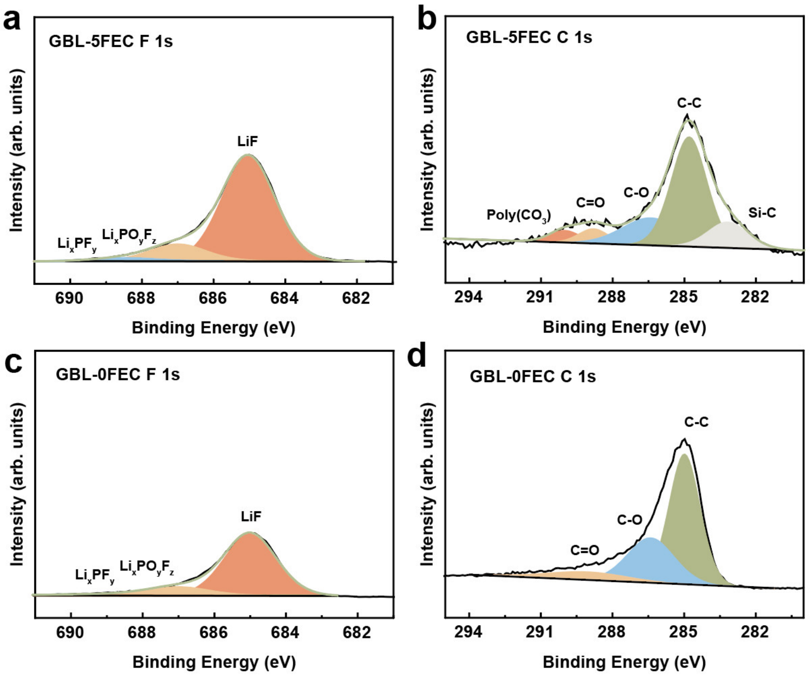

Supplementary Fig. 35. XPS spectra of SD-SEI in GBL-based electrolytes with different FEC contents. The GBL-based electrolytes with different FEC contents were denoted as GBL-mFEC electrolyte, where the "m" represents the volume ratio of FEC. Fig. R8 shows the SD-SEI formed in GBL-based electrolytes with different FEC contents. Obviously, the lowered content of FEC led to the decreased amount of LiF and polycarbonates of SD-SEI in the GBL-5FEC electrolyte and GBL-0FEC electrolyte.

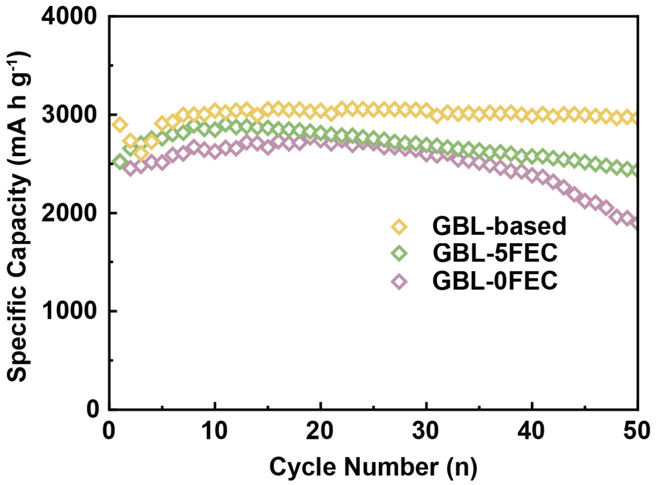

Supplementary Fig. 36. Cycling performance of the micron-sized Si anode in the GBL-based electrolytes with different content of FEC (forming different content of LiF) at 0.2C (1C=3000 mA g^{-1}). The cycling performance of micron-sized Si anode in the GBL-based electrolytes was found to be better than that in the GBL-5FEC electrolyte and GBL-0FEC electrolyte. This result has been summarized in Supplementary Table 4. Therefore, high relative content of LiF in SEI potentially benefits the cycle performance of Si anodes, and the synergistic effect of LiF and polycarbonates improve the cycle performance more significantly.

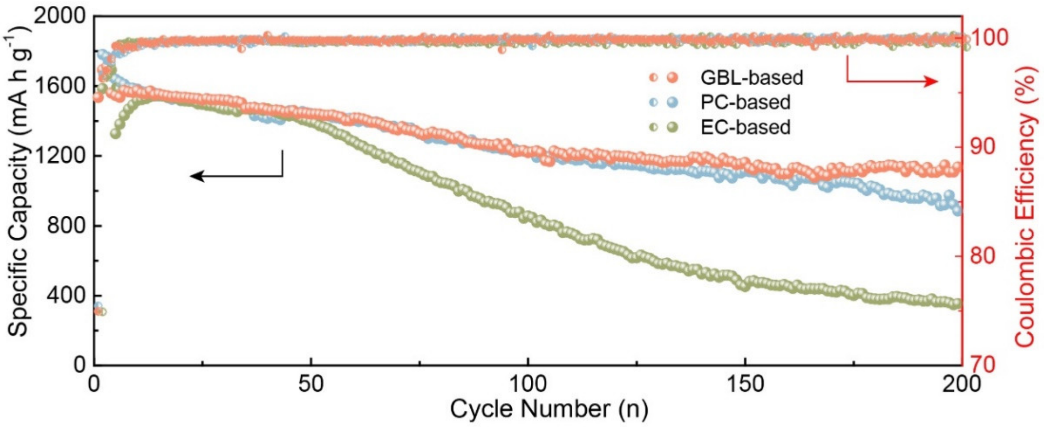

Supplementary Fig. 37. Cycling performance and CEs of Li||SiO_x half-cells cycled in various electrolytes at 0.2C, where 1C=1500 mA g^{-1}.

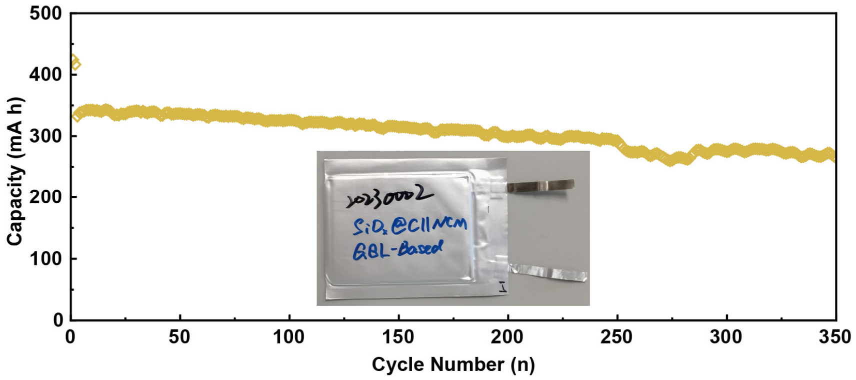

Supplementary Fig. 38. Cycling performance of G-SiO_x||NCM811 pouch cell at current density of 800 mA. The inset shows the optical image of the pouch cell.

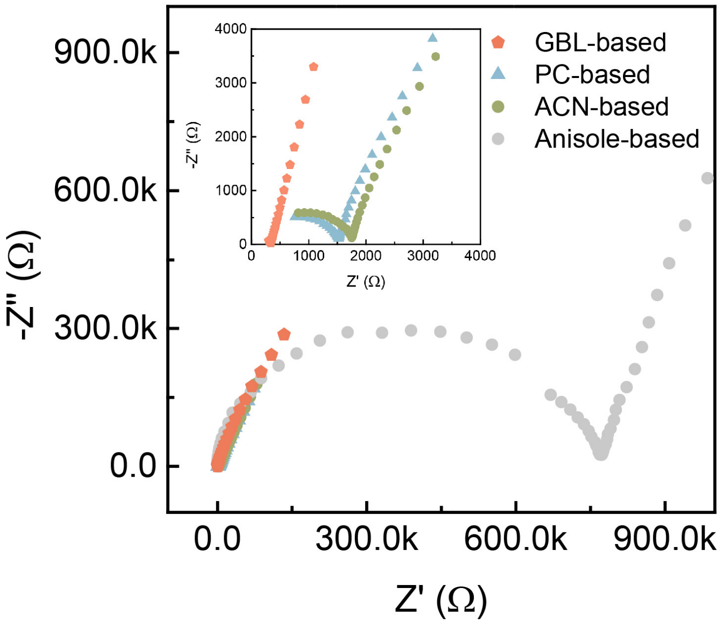

Supplementary Fig. 39. Nyquist plots of SS||SS coin cell with various saturated solution of CH_3CH_2OLi in various electrolytes. The corresponding ionic conductivities of the solution can be figured out according to the equation (1)

$$k = \frac{L}{AR} \quad (1)$$

Where L is the distance of two electrodes, A is the contact area of electrolytes and SS electrode and R is the measured impedance value. Hence, the relative solubilities of these solvents were determined by the resulting ion conductivities, which was plotted in Figure 5c.

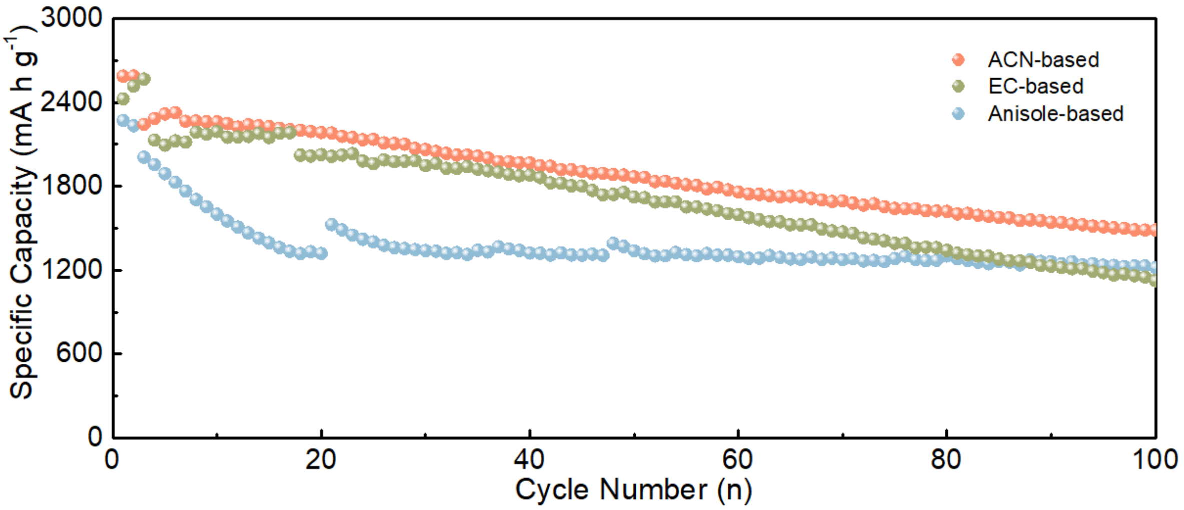

Supplementary Fig. 40. Cycling performance of Li||Si@C half-cells cycled in electrolytes with various polarities at current density of 0.2C, where 1C=3000 mA g^{-1}.

Supplementary Table 1 | Comparison of modulus and elastic stain limit of typical SEI components.

	LiF	polycarbonates	$ROCO_2Li$	ROLi
Modulus (E, GPa)	89.6	<1	<1	<1
Elastic strain limit (ε_Y)	low	high	low	low

Supplementary Table 2 | Fitting parameters from EIS plots in Supplementary Fig. 20.

	Electrolytes	R_s	R_{SEI}	R_{ct}
before cycle	GBL-based	1.3	\	721.1
	PC-based	1.8	\	684.6
	EC-based	2	\	587.5
after cycle	GBL-based	4.6	1.6	21.9
	PC-based	2.9	67.6	147
	EC-based	4	193.5	407

Supplementary Table 3 | Comparison of mechanical properties of SEIs in various reports.

Anode	Thickness (nm)	ε_Y (nm)	E (GPa)	Citation
Si	~24.31	\	~69.2	[12]
Si	25	\	3.7	[13]
Si	24	\	~0.696	[14]
Si	62	\	0.296	[15]
Si	145	\	0.095	
Si	14.5	\	2.4	[16]
Sn (SIBs)	~8	~4	0.355	[17]
Na (SIBs)	~12.9	~10.4	~1	[18]
Si	32.8	13.4	1.5	This work

Supplementary Table 4 | SEI with various component configurations and corresponding cycling performance of Si anodes in GBL-mFEC electrolytes.

Electrolyte	LiF Content (at %)	Polycarbonates Content (at %)	Capacity Retention After 50 Cycles (%)
GBL-0FEC	14.92	1.89	75.2
GBL-5FEC	52.59	5.87	96.6
GBL-based	65.8	6.57	102.2
PC-based	75.5	~0	97.2

Supplementary References

1. Gao, Y. & Zhang, B. Probing the Mechanically Stable Solid Electrolyte Interphase and the Implications in Design Strategies. *Adv. Mater.* **35**, 2205421 (2023).

2. Gao, Y. *et al.* Unraveling the mechanical origin of stable solid electrolyte interphase. *Joule* **5**, 1860–1872 (2021).

3. Chen, J. Electrolyte design for LiF-rich solid–electrolyte interfaces to enable high-performance microsized alloy anodes for batteries. *Nat. Energy* **5**, 386–397 (2020).

4. Tan, J., Matz, J., Dong, P., Shen, J. & Ye, M. A Growing Appreciation for the Role of LiF in the Solid Electrolyte Interphase. *Adv. Energy Mater.* **11**, 2100046 (2021).

5. Park, S. *et al.* Replacing conventional battery electrolyte additives with dioxolone derivatives for high-energy-density lithium-ion batteries. *Nat. Commun.* **12**, 838 (2021).

6. Yang, G. *et al.* Robust Solid/Electrolyte Interphase (SEI) Formation on Si Anodes Using Glyme-Based Electrolytes. *ACS Energy Lett.* **6**, 1684–1693 (2021).

7. Gao, Y. *et al.* Polymer–inorganic solid–electrolyte interphase for stable lithium metal batteries under lean electrolyte conditions. *Nat. Mater.* **18**, 384–389 (2019).

8. Yang, Z. *et al.* In-situ cross-linking strategy for stabilizing the LEDC of the solid-electrolyte interphase in lithium-ion batteries. *Nano Energy* **105**, 107993 (2023).

9. Shin, H., Park, J., Han, S., Sastry, A. M. & Lu, W. Component-/structure-dependent elasticity of solid electrolyte interphase layer in Li-ion batteries: Experimental and computational studies. *J. Power Sources* **277**, 169–179 (2015).

10. Heiskanen, S. K., Kim, J. & Lucht, B. L. Generation and Evolution of the Solid Electrolyte Interphase of Lithium-Ion Batteries. *Joule* **3**, 2322–2333 (2019).

11. Kindt-Larsen, T., B. Smith, D. & Steen, J. J. Innovations in acrylic bone cement and application

equipment. *J. Appl. Biomater.* **6**, 75–83 (1995).

12. Zhang, Q., Xiao, X., Zhou, W., Cheng, Y.-T. & Verbrugge, M. W. Toward High Cycle Efficiency of Silicon-Based Negative Electrodes by Designing the Solid Electrolyte Interphase. *Adv. Energy Mater.* **5**, 1401398 (2015).
13. Kamikawa, Y., Amezawa, K. & Terada, K. Elastic–Plastic Deformation of a Solid Electrolyte Interface Formed by Reduction of Fluoroethylene Carbonate: A Nanoindentation and Finite Element Analysis Study. *J. Phys. Chem. C* **124**, 22488–22495 (2020).
14. Huang, S. In-situ study of surface structure evolution of silicon anodes by electrochemical atomic force microscopy. *Appl. Surf. Sci.* **452**, 67–74 (2018).
15. Li, Y. *et al.* Suppressing Continuous Volume Expansion of Si Nanoparticles by an Artificial Solid Electrolyte Interphase for High-Performance Lithium-Ion Batteries. *Appl. Surf. Sci.* **9**, 8059−8068 (2021).
16. Cao, Z., Zheng, X., Qu, Q., Huang, Y. & Zheng, H. Electrolyte Design Enabling a High-Safety and High-Performance Si Anode with a Tailored Electrode–Electrolyte Interphase. *Adv. Mater.* **33**, 2103178 (2021).
17. Huang, J. *et al.* Nanostructures of solid electrolyte interphases and their consequences for microsized Sn anodes in sodium ion batteries. *Energy Environ. Sci.* **12**, 1550–1557 (2019).
18. Gong, C. *et al.* The role of an elastic interphase in suppressing gas evolution and promoting uniform electroplating in sodium metal anodes. *Energy Environ. Sci.* **16**, 535–545 (2023).
