# Supplementary material for: Tailoring chemical composition of solid electrolyte interphase by selective dissolution for long-life micron-sized silicon anode
Source: Nat Commun. 2023 Nov 9;14:7247. doi: 10.1038/s41467-023-43093-6 (PMC10636032; doi:10.1038/s41467-023-43093-6)
Supplement: Supplementary file 3 — Description of Additional Supplementary Files [file 41467_2023_43093_MOESM3_ESM.pdf]

Description of Additional Supplementary Files

File Name: Supplementary Data 1

Description: The initial and final MD configurations used to simulate the dissolution characterizations in GBL and EC solvents.
